# Treatment with long-acting growth hormone: effectiveness and safety

**DOI:** 10.3389/fendo.2026.1818944

**Published:** 2026-05-19

**Authors:** Livia Basile, Rossella Cannarella, Rosita A. Condorelli, Francesco Frasca, Aldo E. Calogero, Sandro La Vignera

**Affiliations:** Department of Clinical and Experimental Medicine, University of Catania, Catania, Italy

**Keywords:** GH, growth hormone, growth hormone (GH) deficiency, growth hormone deficiency, long-acting GH

## Abstract

Long-acting growth hormone (GH) formulations represent a clinically effective alternative to daily recombinant human GH (rhGH) administration, enabling extended dosing intervals and enhancing patient adherence. This approach is particularly beneficial in the pediatric population, where caregivers frequently manage drug administration and children may be either unaware of its therapeutic benefits or reluctant to undergo daily injections. Various technologies have been utilized to prolong the retention of GH in target tissues, achieving either prolonged drug release or delayed clearance. These formulations have shown efficacy in promoting linear growth in children with GH deficiency, with a safety profile comparable to that of daily rhGH therapy. This narrative review evaluates the existing literature on long-acting GH formulations, focusing on their effectiveness and safety in pediatric patients.

## 1 Introduction

Short stature is defined as a height that falls below two standard deviations (SD) from the average for child’s age, sex, and ethnicity, approximately corresponding to the 3^rd^ percentile (1). Growth is influenced by numerous factors, including race, lifestyle, nutrition, culture, and socio-economic conditions. Due to these variables, the causes of short stature in children can differ considerably between developed and developing countries. It is a condition that recognizes both genetic and/or environmental causes interplaying each other (2).

Short stature is acknowledged as a consequence of four main factors: genetics, constitutional growth delay, early puberty, and health-related issues (3). In the case of genetic factors, the condition is referred to as familial short stature, where individuals reach a height within the target range typical for their family (4). These children have normal growth rates and no delay in bone age. Constitutional growth delay is characterized by a slower growth rate, with affected children showing a delayed bone age (5). Although these children are shorter for their age and experience a delay in puberty, they typically catch up in height during adulthood. This condition can be caused by genetic factors or early childhood malnutrition. In cases of precocious puberty, children experience early sexual maturation, occasionally accompanied by central nervous system dysfunction (6). A familial pattern of early puberty also suggests a potential genetic cause.

Short stature can also result from growth hormone (GH) deficiency (GHD) (3). Overall studies indicate that familial short stature, constitutional growth delay, and GHD are among the most common causes of short stature. Variations in the reported prevalence of short stature across different studies can be addressed to differences in healthcare settings, the criteria used to define short stature, and the characteristics of the study populations (7).

GH, also known as somatropin, is a metabolic hormone produced by somatotropic cells in the anterior pituitary gland in a pulsatile manner (8, 9). It plays a crucial role in lipid, carbohydrate, and protein metabolism, and functions as an acute-phase stress reactant. GH secretion is regulated by multiple factors, including stress, exercise, nutrition, sleep, and feedback mechanisms involving three key transcriptional factors. Hypothalamic growth hormone-releasing hormone (GHRH) stimulates GH production and release, while somatostatin, produced in various tissues, acts as a counter-regulatory factor by inhibiting GHRH, thus maintaining a balance between stimulation and suppression. Additionally, insulin-like growth factor-1 (IGF1) inhibits GH release both directly, by acting on somatotropic cells, and indirectly, by enhancing somatostatin secretion. A third key regulator, ghrelin – primarily secreted by the stomach – stimulates GH release, particularly during fasting or periods of low energy availability. Ghrelin also contributes to the counter-regulatory response to prolonged hypoglycemia, helping to maintain metabolic homeostasis (3).

**Figure 1:**
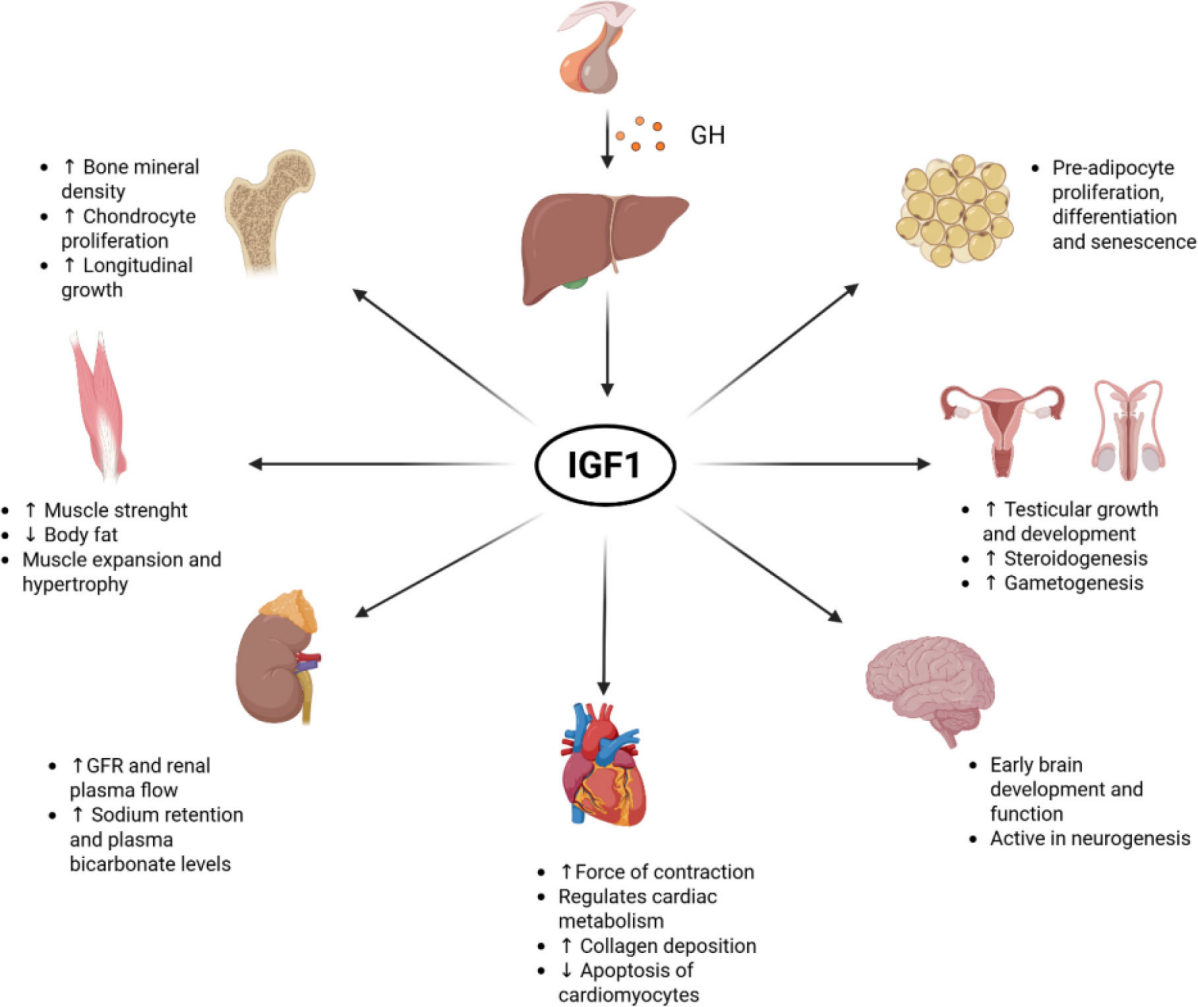
Effects of growth hormone (GH) and insulin-like growth factor 1 (IGF1) on various organs and tissues. GFR, Glomerular filtration rate. Created in BioRender. Calogero, A. (2026).

GH binds to specific cell surface receptors, exerting both direct and indirect effects on various tissues. At the peripheral level, it acts on epiphyseal chondrocytes, while indirectly influencing growth through the activity of IGF1. The combined action of GH and IGF1 play a pivotal role in regulating growth and body composition. However, GH and IGF1 have opposite effects on adipose tissue: GH promotes lipolysis, whereas IGF1 stimulates lipogenesis (10). In the liver, GH counteracts the effects of insulin and stimulates gluconeogenesis (11), contributing to a hyperglycemic state. GH also promotes the growth of all tissues and organs, with a particular emphasis on cartilage and bone during puberty, by enhancing gene expression and cell growth. On the other hand, IGF1 fosters metabolism, anabolism, and cell replication, while inhibiting apoptosis, thereby prolonging the lifespan of existing cells (12) **(****Figure 1**).

These pleiotropic effects of GH are highly relevant in the therapeutic setting. While GH replacement therapy is primarily aimed at promoting linear growth in children with growth hormone deficiency, its actions extend beyond the skeletal system to include significant metabolic and endocrine effects. In clinical practice, GH therapy improves body composition by increasing lean mass and reducing adiposity, and exerts beneficial effects on lipid metabolism, bone mineralization, and cardiovascular function. However, these same mechanisms may also contribute to adverse metabolic outcomes, such as insulin resistance and alterations in glucose homeostasis. Therefore, the systemic effects of GH must be carefully considered when evaluating both the efficacy and safety of different therapeutic formulations, particularly long-acting preparations that result in more sustained hormone exposure.

### 1.1 GH recombinant therapy

Clinically, GH replacement therapy is recommended for the treatment of endogenous GHD in children to stimulate linear growth and increase growth rate (13). GHD may result from decreased hypothalamic or pituitary function, both of which are essential for GH regulation and production, respectively. This condition can be congenital or acquired and may include disorders caused by altered GH activity at the cell level (14).

Human GH (hGH) was first produced in the 1950s by extracting it from cadaveric human pituitaries. However, due to risks of contamination and limitation in supply, it was replaced by recombinant human GH (rhGH) in 1985 for treating children with GHD. Recombinant technology provided an unlimited supply of rhGH, enabling long-term monitoring focused on efficacy, safety, and cost. The availability of rhGH also facilitated the exploration of clinical strategies to optimize administration time and dosage for children with short stature but without GHD. Indeed, rhGH has been approved for use in children with chronic kidney disease, Turner syndrome, Prader-Willi syndrome, and in those born small for gestational age (SGA) who exhibit persistent short stature. Additionally, in the United States and Canada, it is also indicated for children with idiopathic short stature (ISS) (14, 15). Clinical studies have demonstrated the effectiveness of rhGH treatment in children with ISS, showing improvements in height and metabolic benefits without significant side effects (16, 17). Additionally, the long-term safety and efficacy of recombinant human GH therapy in children with GHD have been extensively documented (18).

The effectiveness of rhGH therapy is assessed based on the achieved growth rate, the increase in height, and the serum levels of IGF1. In children with short stature due to GHD, rhGH therapy results in significant catch-up growth. The most rapid growth occurs during the first year of treatment, after which the growth rate slows down and eventually aligns with the normal growth rate of children within a few years. At the same time, body composition normalizes, with increases in muscle mass and strength, alongside reductions in fat mass (19).

GH therapy can have a beneficial effect on growth in children, with reported increases in height SD scores during treatment ranging from approximately 1.8 to 3.5, reflecting catch-up growth rather than final adult height (20). In children with GHD, the amount of GH administered is typically based on the child’s weight or body surface area, with adjustments made to maintain IGF1 at physiological levels (20). The titration of GH levels is carefully managed due to its pulsatile secretion, particularly during stages 3 and 4 of sleep (21). Both levels of IGF1 and IGF binding protein-3 (IGFBP-3) are influenced by GH status and are more stable throughout the day. Nutritional deficiencies can also impact IGF1 levels independently of GH status. GH secretion is continuous in response to pharmacologic secretagogues, such as arginine, clonidine, glucagon, insulin, and L-Dopa (20).

In diagnosing GHD, GH levels should fall below the diagnostic threshold after administration of two different secretagogues. Consistent with its pleiotropic physiological actions, GH therapy exerts effects beyond linear growth, including significant metabolic, cardiovascular, and body composition changes, including positive effects on lipid metabolism, glucose homeostasis, cardiac health, body composition (specifically the balance of muscle mass versus adiposity), and bone mineral density. GHD is associated with dyslipidemia, insulin resistance, hemostatic disturbances, oxidative stress, and chronic inflammation, resembling a metabolic syndrome. By modulating lipid and glucose homoeostasis at the cell and tissue levels, GH exerts both insulin-like and anti-insulin -like effects, decreasing body fat and increasing lean body mass. However, GH therapy also induces insulin resistance and hyperglycemia, promotes lipolysis and lipid oxidation, and inhibits lipogenesis (22).

These pleiotropic effects are particularly relevant when considering different dosing regimens and formulations of GH therapy. Despite the demonstrated efficacy and safety of daily rhGH therapy (23), treatment remains a significant challenge (24). Adherence, defined as “the extent to which a person’s behavior aligns with agreed recommendations from a healthcare provider regarding medication, diet, and lifestyle changes” (25), is often hindered by the burden of daily rhGH injections, which can be problematic for both patients and caregivers and may jeopardize optimal therapeutic outcomes.

In this context, this narrative review with systematic search examines the current literature on the effectiveness and safety of long-acting GH formulations in children.

## 2 Methods

### 2.1 Search strategy

The search strategy combined keywords related to long-acting growth hormone formulations and pediatric populations (e.g., ‘long-acting growth hormone’, ‘somapacitan’, ‘somatrogon’, ‘lonapegsomatropin’, ‘children’, ‘pediatric’). The example search string reported represents the core structure of the search strategy. Searches were performed in the PubMed and Scopus databases, covering articles from their inception through January 2025. Only studies involving human participants were included, with no language restrictions. After the removal of duplicate records, abstracts were evaluated for eligibility.

No controlled vocabulary (e.g., MeSH terms) or formal Boolean expansion strategy was systematically applied, which may have limited the comprehensiveness of the search.

### 2.2 Selection criteria

All eligible abstracts, including those in languages other than English, were retrieved in full and translated into English where necessary. These articles were subsequently assessed for inclusion based on the PICOS framework (Population, Intervention, Comparison/Comparator, Outcome, Study Type) (26) (**Table 1**). Three researchers (L.B., R.A.C., and R.C.) independently conducted the eligibility assessment. Initially, each study’s title and abstract were independently reviewed by two researchers to determine potential inclusion. In cases of uncertainty, the full text was reviewed to reach a final decision. Discrepancies were resolved through discussion, and if consensus could not be achieved, a third reviewer (S.L.V.) made the final determination. Articles meeting the inclusion criteria were then subjected to data extraction.

**Table 1:** Selection criteria of the studies included, based on the PICOS model.

PICOS	Inclusion criteria	Exclusion criteria
Population	Children	Adults, hormonal treatment (other than GH), major comorbidities
Intervention	Long-acting rhGH formulations	–
Comparison	Daily rhGH	–
Outcome	Efficacy parameters Safety parameters	–
Study type	Observational studies, non-randomized trials, RCTs, case series	*In vitro*, animal studies, case reports, communications, proceedings, conference abstracts

rhGH, Recombinant human growth hormone; RCTs, Randomized control trials.

### 2.3 Data extraction

The following data were extracted from the included studies: first author, year of publication, study design, presence of a control group, type of molecule used, administered dose, height velocity, and reported adverse events.

A formal risk-of-bias assessment and certainty-of-evidence evaluation were not performed; therefore, the findings should be interpreted as a qualitative synthesis of the available evidence.

## 3 Results

**Figure 2:**
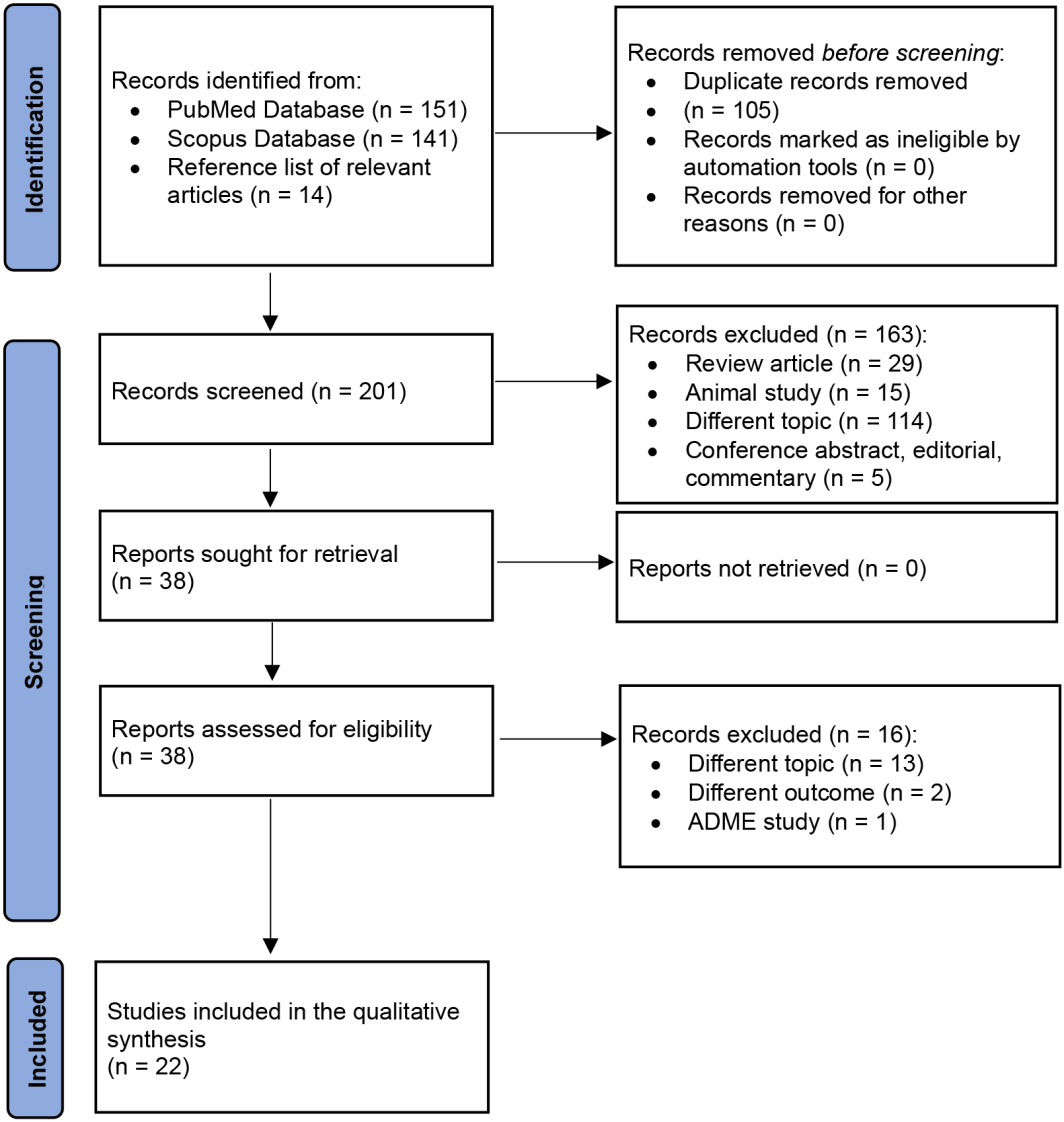
PRISMA 2020 flow diagram of the included studies.

Using the previously described search strategy, 306 abstracts were initially retrieved. After removing duplicates, 201 records were assessed based on title and abstract review. Following the exclusion of 163 abstracts, 38 full-text articles were reviewed for eligibility. Ultimately, 22 articles were included in the final analysis (**Figure 2**). These articles addressed the following products: two focused on somatropin Nutropin Depot (Genentech) (35, 38), four on somatropin LB03002 Eutropin Plus (LG Life Sciences, Ltd.) (34, 61, 62, 63), two on lonapegsomatropin Skytrofa (Ascendis Pharma) (69, 70), two on somatropin Jintrolong (GeneScience Pharmaceuticals Co., Ltd.) (73, 74), eight on somapacitan Sogroya (Novo Nordisk A/S) (39, 44, 45, 46, 48, 49, 50, 51), three on somatrogon OPKO (Health and Pfizer) (55, 57, 58), and one on somavartan VRS-317 (Versartis) (78) (**Table 2**).

**Table 2:** Long-acting recombinant human growth hormone (rhGH) formulation efficacy and safety. outcomes.

Drug	Status	Dose	Efficacy compared with once-daily rhGH	Height velocity (HV) (primary outcome)	Safety outcomes
Nutropin Depot^®^, Genentech					
Silverman et al., 2002 (27)	Discontinued in 2004	1.5 mg/kg once monthly or 0.75 mg/kg twice monthly	Lower	7.2 ± 1.5 cm/year in the first month group and 6.9 ± 1.5 cm/year in the second month group Height standard deviation score (SDS) increased by 1.0 ± 0.5 in the two groups combined	Headache Vomiting Injection site reactions (nodules, erythema, pain, and lipoatrophy)
Reiter et al., 2001 (28)		1.5 mg/kg once monthly or 0.75 mg/kg twice monthly	Lower	HV 7.6 ± 1.7 and 7.9 ± 2.0 cm/year for patients receiving 1.5 mg/kg once monthly and 0.75 mg/kg twice monthly, respectively	Headache Nausea and vomiting Leg pain Injection site reactions (nodules, erythema, pain, and lipoatrophy)
Eutropin Plus™ (LB03002), LG Life Sciences, Ltd.					
Choi et al., 2022 (29)	Approved in Europe and marketed in Korea	0.7 mg/kg/week *versus* once-daily GH 0.37 mg/kg/week	Comparable	HV 8.41 ± 1.06 cm/year *versus* 10.56 ± 0.78 cm/year	No safety issues
Péter et al., 2011 (30)		0.2 mg/kg/wk 0.5 mg/kg/wk 0.7 mg/kg/wk *Versus* Once-daily rhGH 0.03 mg/kg		HV (cm/year): 8.54 ± 0.97 for 0.2 and then 0.5 mg/kg/week HV (cm/year): 9.01 ± 1.33 for 0.5 mg/kg/wk HV (cm/year): 9.30 ± 1.22 for 0.7 and then 0.5 mg/kg/week) HSDS gain from baseline: 2.50 ± 0.74 for 0.2 and then 0.5 mg/kg/week HSDS gain from baseline: 2.43 ± 0.78 for 0.5 mg/kg/week HSDS gain from baseline: 2.67 ± 0.66 for 0.7 and then 0.5 mg/kg/week *Versus* HV (cm/year): 9.18 ± 0.76 for once-daily rhGH 0.03 mg/kg/day and then LB03002 0.5 mg/kg/week (switch) HSDS gain from baseline: 2.64 ± 0.68 for once-daily rhGH 0.03 mg/kg/d and then LB03002 0.5 mg/kg/week (switch)	Pain in extremity Pyrexia Abnormal liver function tests Hypothyroidism GH antibodies Injection site reactions
Khadilkar et al., 2013 (31)		0.5 mg/kg body weight *Versus* 0.03 mg/kg once-daily rhGH		HV (cm/year): 11.63 ± 2.60, Change from baseline, mean ± SD: 8.94 ± 2.91 HV (cm/year) in the second year 8.33 ± 1.92 Change from baseline at 24 months, mean ± SD -3.40 ± 2.41 *Versus* HV (cm/year): 11.97 ± 3.09 Change from baseline, mean SD 9.04 ± 3.19 After switching in the second year 7.28 ± 2.34 Change at 24 months, mean ± SD: -4.88 ± 2.69	Persistent glucose intolerance Positive for antibodies to S. cerevisiae GH antibodies Injection site reactions (mainly swelling, pain, erythema, and discoloration)
Hwang et al., 2018 (32)		0.5 mg/kg/week 0.7 mg/kg/week *versus* 0.37 mg/kg/week once-daily rhGH		LS means for HV change of 5.08 and 3.65 cm/year for 0.5 mg/kg/week, and 0.7 mg/kg/week The LS means for the change in height standard deviation score were 0.65 and 0.49 *versus* LS mean for HV change of 4.38 The LS means for the change in height standard deviation score was 0.58.	Injection site reactions (warmth, erythema, and swelling).
Lonapegsomatropin (Skytrofa), Ascendis Pharma					
Thornton et al., 2021 (33)	Approved by the FDA	0.24 mg/kg/week *versus* 0.034 mg/kg once-daily rhGH	Superior	LS mean (SE) AHV: 11.2 (0.2) cm/year LS mean (SE) height SDS 1.10 (0.04) *versus* LS mean (SE) AHV 10.3 (0.3) cm/year LS mean (SE) height SDS 0.96 (0.05)	Headache Pyrexia Upper respiratory tract infection New onset or worsening deficiencies of other pituitary axes Injection site reactions (urticaria, swelling)
Chatelain et al., 2017 (34)		0.14, 0.21, or 0.30 mg/kg/week *versus* 0.03 mg/kg once-daily rhGH	Comparable	Mean annualized HV 12.9 cm/year for 0.21 mg/kg/week TransCon GH *versus* Mean annualized HV 11.6 cm/year for once-daily rhGH The minimum annualized HV of 6.42 cm/year for 0.14 mg/kg/week *versus* 6.22 cm/year for the once-daily group. The maximum annualized HV of 22.0 cm/year for 0.30 mg/kg/week *versus* 19.25 cm/year for the once-daily group Δheight SDSs increased from 0.7 to 0.9 in the three TransCon GH cohorts compared with 0.6 in the daily rhGH cohort	Nausea and vomiting Mild iron deficiency anemia Injection site reactions (pain)
Jintrolong^®^, GeneScience Pharmaceuticals Co., Ltd.					
Luo et al., 2017 (35)	Marketed in China	0.2 mg/kg/week *versus* 0.25 mg/kg/week once-daily rhGH	Higher	HV 13.41 ± 3.72 cm/year and ΔHeight 1.06 ± 0.40 SDS *versus* HV 12.55 ± 2.99 cm/year and ΔHeight 0.98± 0.32	Headache Hypothyroidism Peripheral edema Injection-site reactions
Qiao et al., 2019 (36)		0.2 mg/kg/week *versus* 0.30 mg/kg/week once-daily rhGH	Comparable	HV 10.08 ± 2.12 cm/year and HtSDS −1.06± 0.85 *versus* HV 9.28 ± 1.22 cm/year and HtSDS −1.13± 0.74	Hypothyroidism
Sogroya^®^ (Somapacitan), Novo Nordisk A/S					
Juul et al., 2023 (3)	Approved by FDA	0.16, 0.20, and 0.24 mg/kg/week *versus* 0.035 and 0.067 mg/kg/day once-daily rhGH	Comparable	Mean annualized HV was 8.9, 11.0, and 11.3 cm/year for somapacitan 0.16, 0.20, and 0.24 mg/kg/week, respectively, Change from baseline in observed mean HVSDS for somapacitan 0.16, 0.20, and 0.24 mg/kg/week:4.03, 6.77, and 7.24 *versus* mean annualized HV 10.3 and 11.9 cm/year for daily GH 0.035 and 0.067 mg/kg/day. Change from baseline in observed mean HVSDS: 6.37 and 7.86 for 0.035 and 0.067 mg/kg/day:	No safety issues
Sävendahl et al., 2023 (37) REAL 3: four years of treatment and 1 year after switching to somapacitan from daily GH		0.04/0.16 mg/kg/week 0.08/0.016 mg/kg/week 0.16/0.16 mg/kg/week *Versus* 0.034 mg/kg/day Once-daily rhGH and Once-daily rhGH 0.034 mg/kg/day somapacitan 0.16 mg/kg/week	Compared	HV (SD) 7.4 (1.6) for somapacitan/somapacitan *versus* 6.6 (1.6) for once daily/GH somapacitan HV SDS change from baseline 4.02 (2.85) for somapacitan/somapacitan *versus* 4.09 (3.17) for once-daily somapacitan Height SDS change from baseline 2.85 (1.25) for somapacitan/somapacitan *versus* 2.28 (0.97) for once daily/GH somapacitan	Injection-site reactions (associated or not to pain)
Miller et al., 2022 (38) REAL 4: 52 week + extension period		0.16 mg/kg/wk *versus* 0.034 mg/kg/d once-daily rhGH	Compared	Mean HV 11.2 Change in HSDS from baseline: 1.25 *Versus* Mean HV 11.7 cm/y for once-daily GH Change in HSDS from baseline: 1.30	Injection site reactions (pain, bruising, hematoma)
Savendahl et al., 2020 REAL 3: 26 and 52 week results (39)		0.04, 0.08, and 0.16 mg/kg/week *versus* 0.034 mg/kg once-daily rhGH	Higher	Mean (SD) annualized HV 8.0 (2.0), 10.9 (1.9), 12.9 (3.5) cm/year, respectively; Mean (SD) change in HV SDS was 4.72 (2.79), 6.14 (3.36), and 8.60 (3.15) *versus* Mean (SD) annualized HV 11.4 (3.3) cm/year once-daily rhGH; Mean (SD) change in HV SDS 7.41 (4.08)	Vomiting Injection-site reactions (edema, hematoma, lipoatrophy)
Miller et al., 2024 (40) REAL 4: two years result after switching		Somapacitan 0.16/0.16 mg/kg/week *versus* Switch 0.034 mg/kg once-daily GH to 0.16 mg/kg/week GH/soma		Annualized HV 8.4 cm/year (1.5) Change in HSDS from baseline 1.8 (0.7) *versus* Annualized HV 8.7 cm/year (1.8) Change in HSDS from baseline 2.0 (1.0)	Injection site reactions (bruising, hemorrhage)
Mori et al., 2024 (41) REAL 4: Japanese patients		0.16 mg/kg/week *versus* 0.034 mg/kg once-daily rhGH	Comparable	Mean HV 10.3 (2.0) cm/year Change from baseline in HVSDS (observed mean (SD): 6.3 (2.3) v*ersus* Mean HV 9.8 (2.6) cm/year Change from baseline in HVSDS (observed mean (SD): 6.3 (3.0)	Injection site reaction
Savendahl et al., 2022 (42) REAL 3 three years results		0.04, 0.08, or 0.16 mg/kg/week *versus* 0.034 mg/kg once-daily rhGH		Change in height velocity SDS from baseline: 5.4 (2.5) for somapacitan 0.04/0.16 mg/kg/wk 4.2 (2.8) for somapacitan (0.08/0.16 mg/kg/wk 5.3 (3.0) for somapacitan (0.16/0.16 mg/kg/wk) 4.9 (2.8) for somapacitan pooled *versu*s Change in height velocity SDS from baseline 5.3 (3.9) for 0.034 mg/kg once-daily rhGH	Abnormal glucose metabolism Injection site reactions (subcutaneous hemorrhage, hematoma, hip deformity, skin atrophy)
Juul et al., 2024 (43) REAL 5: 52 week results		0.16, 0.20 or 0.24 mg/kg/week *versus* 0.035 or 0.067 mg/kg once-daily rhGH	Comparable	HV 8.5, 10.4, and 10.7 cm/year for somapacitan 0.16, 0.20 and 0.24 mg/kg/week, respectively Mean change from baseline in HVSDS: 3.81, 6.17 for 0.16, 0.20, 0.24 mg/kg/week *versus* HV 9.3 and 11.2 cm/year Mean change from baseline in HVSDS: 0.067 mg/kg/day, 7.11 GH 0.035 and 0.067 mg/kg/day: 5.30 and 7.60)	Injection-site reactions
Somatrogon, OPKO Health and Pfizer					
Nataliya et al., 2017 (44)	Approved in Europe and in Canada	0.25, 0.48, or 0.66 mg/kg/week *versus* 0.24 mg/kg/week once-daily rhGH	0.66 mg/kg/week more pronounced response	Mean annualized HV 10.4± 2.6, 11.0 ± 2.3 cm/year for 0.25 and 0.48 mg/kg/week 11.9 ± 3.5 cm/year for 0.66 mg/kg/week *versus* Mean annualized HV 12.5 ± 2.1 cm/year	Injection-site reactions (pain, erythema, hematoma, swelling)
Horikawa et al., 2022 (45)		0.66 mg/kg/week *versus* 0.25 mg/kg/week once-daily rhGH	Comparable	LS mean for HV was 9.65 ± 0.97 cm/year ΔHeight 0.94 ± 0.23 SDS *versus* LS mean for HV 7.87 ± 2.61 cm/year ΔHeight 0.52 ± 0.61 SDS	Pyrexia Nasopharyngitis Pharyngitis Influenza Injection-site pain
Zadik et al., 2023 (46) OLE		0.25 mg/kg/week 0.48 mg/kg/week 0.66 mg/kg/week		Mean (SD) annual HVs: 7.73 (1.89) cm/year and 7.54 (1.28) cm/year for 0.25 and 0.48 mg/kg/week, respectively The mean (SD) annual HV: 8.81 [1.12] cm/year for 0.66 mg/kg/week cohort Height SDS at the end of year 1 of OLE: −2.06 (0.85) Height SDS at the end of year 2–4 of OLE: −1.59 (0.80) and −1.27 (0.93) Height SDS at the end of PEN: −0.69 (0.87)	Scoliosis Mild hypercholesterolemia Injection site reactions (bruising in two participants and erythema in one participant)
Somavartan (VRS-317) Versartis					
Moore et al., 2016 (47)	Discontinued in 2017	1.15 mg/kg/week 2.5 mg/kg/biweekly 5 mg/kg/monthly	Lower	HV 7.9 ± 2.5 cm/year (1.15 mg/kg/week) HV 8.6 ± 2.7 cm/year (2.5 mg/kg/biweekly) HV 7.6 ± 2.5 cm/year (5 mg/kg/monthly)	Headache, Decreased free T_4_ Dizziness Increased blood glucose Injection-site reactions (pain, erythema) Urticarial rash

HV, Height velocity; LS, Least square; SDS, standard deviation score; OLE Open-label extension; PEN, Somatrogon delivery via prefilled.

### 3.1 Long-acting growth hormone formulations

Long-acting GH formulations offer a clinically effective alternative to daily rhGH therapy by extending dosing intervals and enhancing patient adherence. This is especially important in the pediatric population, where caregivers are often responsible for administering the treatment, and children may be either unaware of its therapeutic benefits or reluctant to undergo daily injections.

The development of long-acting GH formulation began in 1999, with several pharmaceutical companies subsequently creating formulations that provide sustained GH release on a weekly, biweekly, or monthly basis. These depot formulations were designed to control serum GH levels and manage *in vivo* clearance (48). Long-acting GH products have demonstrated efficacy in promoting linear growth in children with GHD, with a safety profile comparable to daily rhGH therapy (49). Moreover, poor adherence to daily rhGH injections has been shown to negatively impact treatment outcomes, as evidenced by height data from children undergoing replacement therapy who fail to achieve average population height. In children and adolescents, adherence to GH therapy typically declines over time, resulting in a temporary decrease in height velocity (HV) and serum IGF1 levels. Long-acting GH formulations, by extending the GH half-life and reducing injection frequency, are thought to improve adherence, particularly in patients who are reluctant to undergo daily rhGH injections, thus enhancing clinical outcomes. From both a quality of life and cost-effectiveness perspective, long-acting GH formulations, may offer a viable alternative in GHD therapy (50, 51).

Various technologies have been developed to extend the pharmacokinetics of GH, providing prolonged drug release or delayed clearance. These approaches can be broadly classified into the following categories:

1. Depot formulations: These are designed to allow slow diffusion of GH from a polymeric matrix into surrounding tissues and vasculature. Examples include somatropin formulations such as Nutropin Depot and LB03002 (Eutropin Plus), which use microsphere-based delivery systems or biodegradable polymers to achieve sustained release.
2. PEGylated formulations: The conjugation of GH with polyethylene glycol (PEG) increases its hydrodynamic size, thereby prolonging circulation time and reducing renal clearance. Examples include Jintrolong and Lonapegsomatropin (based on TransCon technology).
3. Albumin-binding GH analogues: These compounds are modified by the addition of fatty acid moieties that enable reversible binding to serum albumin, thereby extending half-life. Somapacitan is a representative example of this approach.
4. Fusion proteins: In this strategy, GH is fused with other proteins, such as fragments of human immunoglobulin or other carrier molecules, to delay clearance. An example is Somatrogon, which is a GH fusion protein.

**Figure 3:**
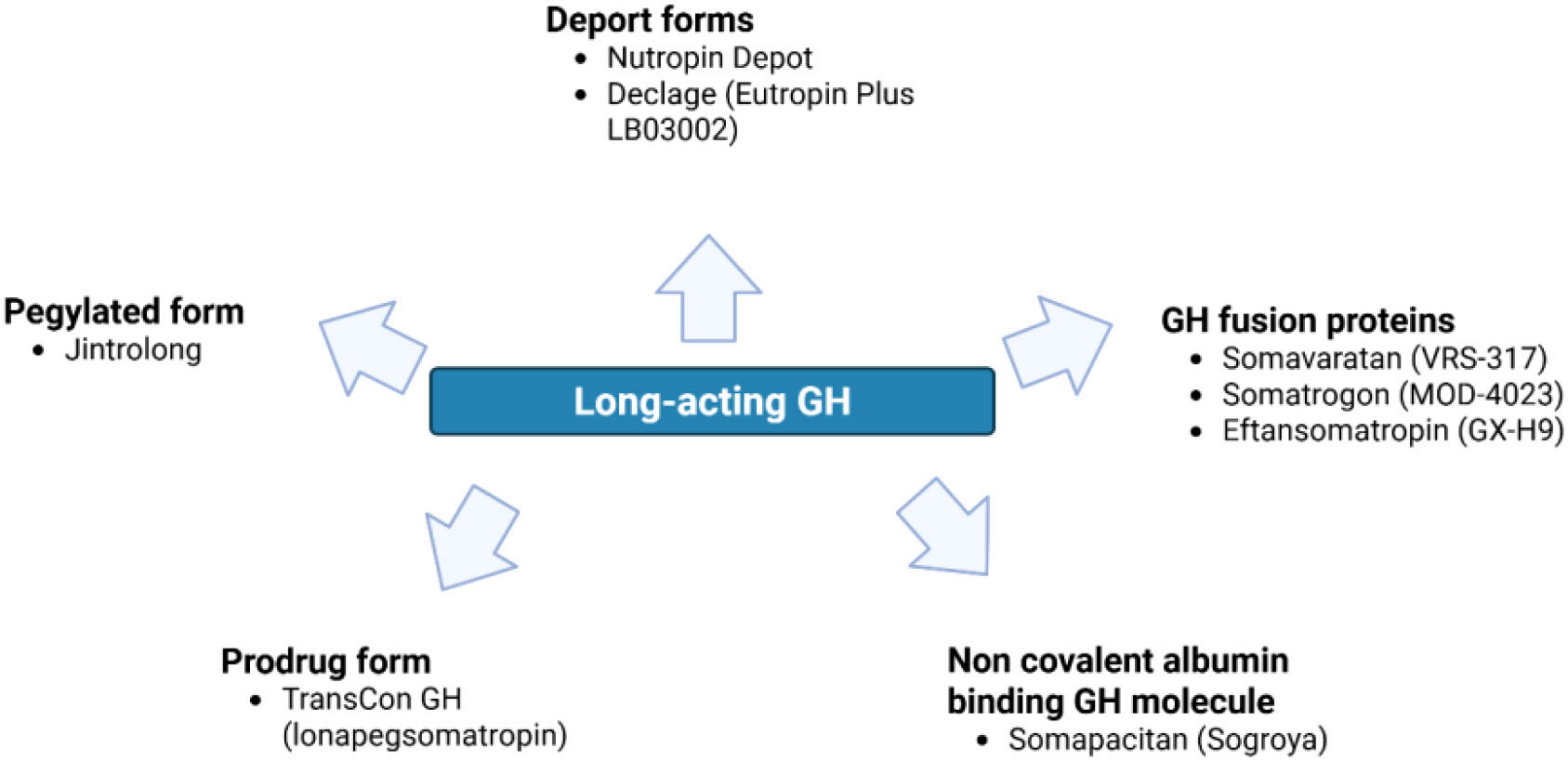
Long-acting growth hormone (GH) formulations. Created in BioRender. Calogero, A. (2026).

Overall, these modifications—whether through depot systems, PEGylation, albumin binding, or protein fusion—are designed to maintain therapeutic GH levels for extended periods, thereby reducing injection frequency while preserving efficacy (52) (**Figure 3**).

A meta-analysis has reported significant reductions in cholesterol and LDL cholesterol levels following GH treatment (52). However, prolonged exposure to sustained elevated GH and IGF-1 levels with long-acting GH formulations may theoretically influence endogenous GH secretion in patients with non-GHD short stature, although clinical evidence supporting this effect remains limited. Evidence from studies on daily recombinant human GH (rhGH) suggests that GH therapy may lead to insulin resistance, although the underlying mechanisms remain unclear. Clinical trials in children with GHD receiving daily rhGH have reported increased insulin resistance, while fasting glucose, postprandial glucose, and HbA1c levels typically remain within normal ranges. Data on long-acting GH formulations are still limited, and their long-term metabolic effects require further investigation (53).

Overall, treatment with either long-acting GH or daily rhGH preparations in children with idiopathic short stature did not significantly affect lipid or carbohydrate metabolism. Data on fasting glucose, HbA1c, fasting insulin, HOMA-IR, as well as triglyceride, total cholesterol, LDL cholesterol, and HDL cholesterol, showed no significant changes following treatment with long-acting GH or high-dose daily rhGH (29).

The following sections of this article will provide a comprehensive review of the various long-acting GH formulations (**Figure 3**) that have been developed and for which published data are available. This review will cover their pharmacokinetic properties as well as the clinical evidence supporting their efficacy and safety in treating patients with GHD.

#### 3.1.1 Depot-formulated somatropin

Depot-formulated somatropin, produced using recombinant DNA technology, is a sustained-release rhGH formulation available as an injectable suspension (Nutropin Depot^®^, Genentech, San Francisco, Ca, USA). Approved by the US Food and Drug Administration (FDA) in 1999, it was the first long-acting GH formulation authorized for subcutaneous (s.c.) administration, with dosing options of either once or twice monthly (27). In this formulation, unmodified GH is encapsulated within a matrix of poly D/L lactide co-glycolide co-polymers, resulting in a biocompatible, biodegradable suspension of microspheres. Upon s.c. injection, GH is gradually released through diffusion from the surface of microsphere into the surrounding tissue over a period up to one month. Meanwhile, the polymer undergoes hydrolysis, breaking down into lactic and glycolic acids, which are ultimately degraded to CO_2_ and water.

Pharmacokinetic and pharmacodynamic parameters following single or multiple doses of GH revealed that serum IGF1 levels increased within the first 14 to 17 days, with the sustained slow release of GH continuing for up 60 days. These data are derived from three studies, involving 138 children treated with GH once or twice monthly (54). Clinical trials have shown that GH improves growth rate and increases standardized height measurements in children with GHD.

The efficacy and safety of GH were evaluated in a 24-month trial involving 56 previously untreated, prepubertal children with GHD. Two dosing regimens were tested: 1.5 mg once monthly (once monthly) and 0.75 mg every two weeks (twice monthly). While no significant difference in growth rate was observed between the two groups at either 12 months (8.3 ± 1.5 cm/year for the once-monthly group and 8.2 ± 2.0 cm/year for the twice-monthly group) or 24 months (7.2 ± 1.5 cm/year for the once-monthly group and 6.9 ± 1.5 cm/year for the twice-monthly group), both rates were lower than those typically seen with daily rhGH injections (27).

The slower skeletal growth progression after 24 months of treatment, with GH, as well as the reduced growth rate, may be attributed to lower GH exposure compared to daily rhGH injections (typically 0.3 mg/kg/week). Despite these differences, children treated with GH showed improvements in growth rate and standardized height measurements, with a mean increase of 1.0 SD in height after two years. Safety data indicated that the s.c. administration was generally well tolerated, with the most common adverse events (AEs) being pain and nodules at the injection site, though these did not lead to treatment discontinuation. Headache and vomiting were reported in fewer than 5% of patients (27).

The initial clinical experience with GH in children was reported in two multicenter, open-label, parallel-group clinical trials (Phase I/II and III studies). The first study provided data on safety and efficacy, using three different doses of this long-acting GH formulation (55). The second study, conducted by Reiter and colleagues (28), reported significant improvements in growth rate, standardized height, and predicted adult height, along with positive feedback from both patients and caregiver. This 6-month open-label trial assessed the safety and efficacy of s.c. administration of GH. The results showed a significant increase of growth rate from baseline. Growth rates were comparable between the 1.5 mg/kg once-monthly or 0.75 mg/kg twice-monthly injection groups, with mean SD growth rate of 8.4 ± 2.1 cm/year at 6 months and 7.8 ± 1.8 at 12 months, compared to 4.5 ± 2.3 cm/year at baseline.

Additionally, the mean bone age advanced by 1.0 year during the first year of treatment with GH, suggesting no undue acceleration of skeletal maturation. This advancement was consistent across both dosage groups. The study tracked growth data from 69 patients who completed 6 months and 56 patients who completed 12 months of treatment in an extension study. Consistent with the results of the study by Silverman and colleagues (27), the average growth rate in this study was lower than those observed with daily rhGH injections. These differences may be attributed to the higher severity of GHD in studies involving daily rhGH injections, which likely led to more pronounced initial catch-up growth compared to long-acting GH.

Evidence on GH treatment indicates an inverse relationship between growth rate, age, and maximum GH levels. In younger patients with more severe GHD, the growth response to GH were similar to that observed in patients using daily rhGH. However, discrepancies in growth responses were noted, potentially due to lower GH and IGF1 levels in patients using GH. Pharmacokinetic data on the two dosing regimens revealed that the bioavailability of GH was dose-dependent, particularly during the initial release phase. Within the first two days after administration, approximately 50-60% of GH is released from the depot, with the remaining amount gradually released over the following days, without prolonged exposure on a monthly basis. Although the depot formulation contains a higher dose than daily rhGH, it results in lower bioavailability. This was attributed to the reduced aggregate GH exposure in children using GH compared to those receiving daily GH injections. This difference in GH exposure may have significant implications, such as the development of insulin resistance and glucose intolerance. Regarding treatment discontinuation, the analysis showed a slight decline in growth rate in the 1.5 mg/kg once-monthly group. However, no significant differences in growth rate were observed in the twice-monthly group related to treatment discontinuation. The first long-acting GH formulation, Nutropin Depot, was withdrawn from the market in 2004, primarily due to manufacturing and commercialization challenges, although injection-site adverse events were relatively common.

#### 3.1.2 Somapacitan

Somapacitan (Sogroya^®^ Novo Nordisk A/S, Copenhagen, Denmark) is a long-acting GH analog that maintains 99% of the structure of endogenous human GH. A substitution of leucine with cysteine at position 101 in the GH backbone enables the attachment of an albumin-binding moiety, which consists of a C16 fatty acid and a hydrophilic spacer conjugated to cysteine at position 101. This modification allows the derivative to bind non-covalently to serum albumin, with fatty acid moiety facilitating reversible binding. This binding mechanism delays clearance and prolongs molecule’s half‐life.

Somapacitan has been approved by the US FDA for the treatment of short stature in children born for SGA (56). However, for somapacitan to be considered a valid alternative to daily rhGH injections, its safety profile must be thoroughly assessed, particularly in terms of GH and IGF1 accumulation. Elevated serum IGF1 levels have been associated with an increased prevalence of AEs, particularly those related to fluid retention and impaired glucose metabolism.

In a pooled model analysis (57) of three phase I trials, pharmacokinetic and IGF1 data were collected from both adults and children with GHD who received once-weekly somapacitan at various doses. The studies included adults with GHD, who received once-weekly single or multiple doses of 0.02, 0.04, 0.08, and 0.12 mg/kg somapacitan (58), adults without GHD who received doses of 0.02, 0.04, 0.08, and 0.12 mg/kg once weekly (59), as well as children with GHD, who received single doses of 0.02, 0.04, 0.08, and 0.16 mg/kg (60). Pharmacokinetic and pharmacodynamic profiles were predicted using non-linear pharmacokinetic modeling and serum IGF1 levels.

The authors suggested that the observed differences in pharmacokinetics and pharmacodynamics between children and adults may be due to body weight, which was identified as a covariate influencing IGF1 response and showing an inverse relationship to pharmacokinetics. The analysis revealed elevated serum IGF1 levels from baseline throughout the dosing interval, with levels not exceeding +2 SD scores when compared to daily rhGH administration. Furthermore, somapacitan demonstrated minimal or no accumulation after once-weekly injections in both children and adults with GHD, supporting the use of a weekly dosing regimen (0.04–0.16 mg/kg) in children with GHD (57). These findings helped to justify the initiation of further clinical trials of somapacitan, including the REversible ALbumin binding (REAL) 3 (Phase 2) (37) and REAL 4 (Phase 3) (38) studies, which explored the safety and efficacy of somapacitan in children with GHD.

REAL 3 is a multicenter, randomized, controlled, double-blind, Phase 2 study conducted in two phases: a 26-week main phase and a 26-week extension phase (NCT02616562) (39). The study aimed to evaluate the efficacy, safety, and tolerability of once-weekly somapacitan versus daily rhGH in children with GHD. In this study, 59 GH treatment-naïve prepubertal children from 11 countries were randomly assigned to receive one of three doses of somapacitan (0.04, 0.08, or 0.16 mg/kg/week) or daily rhGH (0.034 mg/kg/day), all administered s.c. The results showed that after 26 weeks, the 0.16 mg/kg/week dose of somapacitan demonstrated similar efficacy to daily rhGH, with a mean (SD) annualized HV of 12.9 (3.5) cm/year in the somapacitan group compared to 11.4 (3.3) cm/year in the daily rhGH group. By week 52, HV was sustained and significantly greater in the somapacitan 0.16 mg/kg/week group compared to daily rhGH, with a mean (SD) change from baseline in HV SD score of 8.60 (3.15) for the somapacitan groups versus 7.41 (4.08) for the daily rhGH group. Safety and tolerability profiles were consistent with those observed with daily rhGH after 26 and 52 weeks of treatment.

In the Phase 3 REAL 4 trial, once-weekly somapacitan at a dose of 0.16 mg/kg/week was shown to be non-inferior to daily rhGH treatment (Norditropin^®^ at a dose of 0.034 mg/kg/day) in terms of efficacy, safety, and tolerability over a period of 104 weeks in prepubertal, treatment-naïve children with GHD (58). Data from somapacitan Phase 1 trials (58–60) and the REAL 3 study group (39) demonstrated that a 0.16 mg/kg/week dose of somapacitan closely mirrors daily rhGH injections in terms of efficacy, safety, and tolerability in both children and adults with GHD. Following somapacitan administration, the mean peak and trough IGF1 SD score levels were within the physiological range, measuring +1.66 and −0.83, respectively. The highest IGF1 SD score levels were observed three days after administration, with levels decreasing to their lowest point on the seventh day. In daily rhGH treatment, mean IGF1 SD score levels typically range from −2 to +2. While levels exceeding this range should generally be avoided, short-term IGF1 SD score levels above +2 are not typically associated to safety concerns (61).

In terms of growth outcomes in the REAL 4, participants receiving somapacitan showed similar height measures to those receiving daily rhGH (Norditropin^®^), with an estimated mean HV after 52 weeks of treatment (the primary endpoint) of 11.2 cm/year for somapacitan and 11.7 cm/year for daily rhGH. Additionally, the mean change in height SD score and height-for-age SD score were 8.05 and 1.25, respectively. The safety profile of somapacitan was comparable to that of daily rhGH, with no significant differences in AE, immunogenicity, metabolic complications, tolerability issues, or injection site reactions. Furthermore, both somapacitan and daily rhGH treatment similarly reduced the impact of GHD on physical functioning, emotional well-being, and social well-being, as assessed by the Growth Hormone Deficiency—Child Impact Measure (GHD-CIM). Both treatments reduced the disease burden to a similar degree. Despite the comparable effectiveness of both treatments, the once-weekly administration of somapacitan significantly reduced the number of injections (52 per year for somapacitan versus 365 per year for daily rhGH), translating into a reduction in treatment burden for both patients and caregivers, as evidenced by the statistically significant improvement in Growth Hormone Deficiency—Parent Treatment Burden (GHD-PTB) scores.

Beyond the 52-week main phase of the REAL 4 trial, the efficacy of somapacitan persisted throughout the 3-years safety extension period (40). The safety profile remained consistent for both groups: those who continued with once-weekly somapacitan and those who switched from daily rhGH to somapacitan (41). Notably, injection site AEs reported between weeks 52 and 104 were minimal in both groups, and no patients experienced pain at the injection site during the second year of treatment. According to the Growth Hormone-Patient Preference Questionnaire (GH-PPQ), both patients and caregivers strongly preferred once-weekly somapacitan over daily rhGH, particularly because of a lower number of injections and less concern about remembering to administer injections. These advantages, along with a low percentage of injection site AEs (2.3% in the somapacitan-to-somapacitan group and 2.9% in the switch group), likely contributed to improved adherence to once-weekly somapacitan compared to daily rhGH.

These findings are consistent with those from the Phase 2 REAL3 trial, which demonstrated the continued efficacy and safety profile of somapacitan (0.16 mg/kg/week) over a 3-year treatment period in prepubertal, GH‐naïve children with GHD, compared to daily rhGH injections (0.034 mg/kg/day) (42). Furthermore, the preference for once-weekly somapacitan over daily rhGH was also observed in patients and caregivers who switched from daily rhGH treatment during the fourth year of the REAL3 trial (37).

REAL 5, a randomized, multi-center, open-label, controlled Phase 2 study, is the first study to investigate the efficacy, safety, and tolerability of once-weekly somapacitan in children born SGA, compared to daily rhGH. This trial consisted of three phases: a 26-week main phase, a 26-week extension phase, and an ongoing 4-year safety extension. The study involved 62 GH-treatment-naïve, prepubertal short children born SGA, who were randomly assigned to receive s.c. somapacitan (0.16, 0.20, or 0.24 mg/kg/week) or daily rhGH (0.035 or 0.067 mg/kg/day). After 26 weeks of treatment, data from height-based outcomes and with exposure-response analysis showed that somapacitan 0.24 mg/kg/week was the most effective, with similar efficacy, safety, and tolerability as daily rhGH 0.067 mg/kg/day in these children (41). Moreover, after 52 weeks of treatment, the dose-dependent growth response for somapacitan was sustained (43).

#### 3.1.3 Somatrogon

Somatrogon (OPKO Health, Miami, FL, USA, and Pfizer, New York, NY, USA) is a 47 kDa fusion protein produced using carboxy-terminal peptide technology, which involves attaching the 28 naturally occurring carboxy-terminal residues of human chorionic gonadotropin to GH. This modification extends the protein’s half-life (62–64). It has been approved for the treatment of pediatric growth hormone deficiency in Europe, Canada, the United States, and Japan, but not for adult GHD.

A clinical trial involving adults with GHD demonstrated that once-weekly administration of somatrogon was as effective as daily rhGH (Genotropin^®^, Pfizer, New York, NY, USA) (45). Clinically, it has also been developed as a once-weekly treatment for children with growth hormone deficiency (GHD) (65). In a multicenter, open-label, randomized, controlled Phase 2 study, the safety, efficacy, and tolerability of somatrogon were assessed in prepubertal children with GHD. This trial compared three different weekly doses of somatrogon (0.25, 0.48, or 0.66 mg/kg/week) over a 12-month period with daily rhGH (0.24 mg/kg/wk) (44). The results showed that the somatrogon had a longer half-life than daily rhGH. However, the discrepancy in half-life after somatrogon administration was primarily attributed to intrapatient variability and the small sample size within each cohort. During the study, IGF1 SD score levels increased in most patients, except for those in the lowest dose group (0.25 mg/kg/week), which experienced a rapid decline in IGF1 SD score to suboptimal levels. This decline was likely due to the dose, which failed to maintain optimal serum IGF1 levels during somatrogon treatment. Preclinical studies had suggested that somatrogon might be less effective than rhGH (62). However, the growth indexes (HV and ΔHt SD scores) observed after 12 months of somatrogon treatment were comparable to those seen in the normal, age-matched population, with most patients demonstrating an accelerated growth rate compared to age-matched controls. Among all three dose cohorts, the 0.66 mg/kg/week group exhibited the most favorable outcomes in terms of HV, HV SDS, and ΔHt SD score, closely resembling the results obtained with daily rhGH therapy. Regarding safety, somatrogon was well tolerated by all patients throughout the 12-month treatment period, with no serious AEs. The frequency of AEs did not significantly differ across the escalating doses of somatrogon. Overall, the nature and the severity of AEs were consistent with those reported for daily rhGH therapy. One case of mild adrenal insufficiency and one case of moderate secondary adrenocortical insufficiency were reported, both of which were likely related to the drug injection. These cases may have been unmasked by the GH treatment, which has the potential to reveal previously undiagnosed or subclinical adrenocortical insufficiency. The one-year study was extended into a five-year, open-label analysis with five study periods to further assess the safety and efficacy of somatrogon (46). Data from this extended trial demonstrated that long-term somatrogon administration resulted in sustained improvement in growth outcomes, including annual HV, height SD score, and change in height SD score. Participants who initially received on daily rhGH (Genotropin^®^, Pfizer, New York, NY, USA) for 12 months and were subsequently switched to somatrogon also continued to experience linear growth throughout the trial. Mean height for age and gender in participants receiving once-weekly somatrogon treatment remained consistent with the data from the main study (44), with height SD score approaching zero during Periods III to V). Participants receiving the lowest doses (0.25 and 0.48 mg/kg/week) demonstrated similar increases in mean height SD score and change in height SD score across all trial periods (III, IV, and V). However, in the 0.66 mg/kg/week cohort, greater improvements in mean height SD score and more pronounced variations in height SD score ​​were observed. The administration of once-weekly somatrogon over five years in this open-label study was comparable to the main study (44) in terms of prevalence and types of AEs. The main study (44) demonstrated that somatrogon was well tolerated by children with GHD, including those who switched to the pen device for administration. These findings, along with the high adherence rate reported (>90%) throughout the five-year open-label period, suggest that once-weekly somatrogon could provide a valuable alternative for patients with GHD. This regimen has the potential to enhance treatment adherence, a critical factor in optimizing treatment efficacy. Low compliance or early discontinuation of therapy in GHD patients often results in a gradual decline in growth and therapeutic response (66). Furthermore, it has been reported that long-acting GH treatments, such as somatrogon, cause greater fluctuations in serum IGF1 levels compared to daily rhGH treatments. However, the authors did not clarify whether these larger oscillations in IGF1 levels after dosing are attributable to metabolic changes related to adiposity or other factors. Pharmacokinetic and pharmacodynamic analysis of somatrogon revealed that the mean IGF1 SD score was most accurately measured four days after a one-week dose across the dosing range. While IGF1 levels measured 2–3 days after somatrogon injection corresponded to peak IGF1 SD score, these levels were short-lived and declined over the dosing interval. This pattern of increase and decrease mirrors the physiological growth process, where significant rise in IGF1 levels are observed during puberty’s growth spurt (67). Indeed, mean IGF1 levels measured four days after somatrogon injection provide a more effective measure of safety, as they reflect total systemic exposure to IGF1 levels.

#### 3.1.4 LB03002

LB03002 (EutropinPlus injection, Egrifta, LG Chem, Seoul, South Korea) is a once-weekly long-acting GH produced from genetically modified *Saccharomyces cerevisiae* in sodium hyaluronate microparticles suspended in oil base of medium-chain triglycerides prior to injection. This depot form of rhGH has been proven to be both effective and safe in children and adults with GHD (30–32). Furthermore, a study involving the administration of once-weekly LB03002 to Korean prepubertal children with idiopathic short stature for one year showed that daily rhGH did not interfere with the regular, intermittent secretion of GH (68). This remained true even after 12 months of treatment with either long-acting GH (0.7 mg/kg/week) or daily rhGH (0.37 mg/kg/week), both of which did not disrupt normal endogenous GH production (29). LB03002 has been approved in Europe and is marketed in Korea for the treatment of children with GHD. After 12 months of treatment with long-acting GH in patients with idiopathic short stature, both elevated GV and height SD score were observed (29). The efficacy and safety profiles of long-acting GH and daily rhGH therapies were comparable. Specifically, in the long-acting GH group, HV increased from 5.23 ± 1.07 cm/year at baseline to 8.41 ± 1.06 cm/year, while in the daily rhGH group, HV increased from 5.78 ± 0.78 cm/year at baseline to 10.56 ± 0.78 cm/year. Height SD score increased from −2.43 ± 0.29 at baseline to −1.73 ± 0.33 at month 12 in the long-acting GH group, and from -2.43 ± 0.21 at baseline to -1.30 ± 0.28 at month 12 in the daily rhGH group. According to the authors, discontinuation of GH treatment in children who are not GH-deficient but have idiopathic short stature is unlikely to pose a significant risk of growth deceleration. In this study, metabolic outcomes, including fasting glucose, HbA1c, fasting insulin, HOMA-IR levels, triglycerides, total cholesterol, LDL cholesterol, and HDL cholesterol, remained unchanged following treatment with either long-acting GH or high-dose daily rhGH. These findings suggest that both formulations did not have a negative impact on carbohydrate or lipid metabolism. Additionally, measurements of serum thyroid hormone levels during GH treatment showed values within the physiological range. However, in patients receiving daily rhGH, TSH levels at month 6 were lower compared to baseline.

The prevalence of AEs was comparable between the two groups (29).

#### 3.1.5 PEGylated growth hormone formulations

PEGylation, the covalent attachment of PEG to peptides, proteins, drugs, and bioactive compounds, is a widely used technique to extend the circulation time of drugs in the body (69–71). By increasing the molecular size of the drug, PEGylation reduces the likelihood of renal filtration and enzymatic degradation, leading to a more prolonged release of the active compound into circulation. This results in a sustained therapeutic effect (72). Additionally, the PEG layer serves as a protective barrier, shielding the drug from immune system recognition and minimizing the risk of adverse reactions. As such, PEGylated drugs are particularly beneficial for conditions requiring long-term treatment, such as hormone deficiencies. Depending on the formulation, PEG can either be permanently attached or linked via a cleavable bond, enabling controlled drug release (72). PEGylated prodrugs are designed to release their active drug in response to specific physiological conditions. These prodrugs utilize stimuli-responsive PEG linkers that degrade or undergo structural changes under targeted conditions, ensuring controlled and site-specific drug release.

Among PEGylated GH formulations, compounds such as PEGylated somatropin have demonstrated efficacy in increasing growth velocity and maintaining IGF-1 levels within the target range in children with GHD in clinical trials. However, despite these promising results, some PEGylated GH formulations have not been approved in certain regions, including Europe and the United States. This is mainly due to regulatory concerns regarding the potential long-term accumulation of PEG in tissues and the limited availability of long-term safety data, particularly in pediatric populations. Consequently, further studies are required to better define their long-term safety profile.

##### 3.1.5.1 Lonapegsomatropin

Lonapegsomatropin (Skytrofa, Ascendis Pharma, Copenhagen, Denmark) is an inactive prodrug of GH obtained by attaching unmodified rhGH to a methoxypolyethylene glycol carrier molecule through the self-cleaving TransCon (Transient Conjugation) Linker. The linker cleavage, which is dependent on pH and temperature, allows for the prolonged presence of GH in the bloodstream, ensuring a sustained release of rhGH (34). Over a one-week period, hydrolysis of the TransCon Linker at physiologic pH and temperature facilitates the controlled release of unmodified GH. Lonapegsomatropin has been approved for use in the United States.

This GH derivative was evaluated in a randomized Phase 2 study involving prepubertal children with GHD, who received lonapegsomatropin at three different weekly doses (0.14, 0.21, or 0.30 mg/kg/week). The objective of the trial was to compare the pharmacokinetics, pharmacodynamics, safety, and efficacy of weekly lonapegsomatropin to daily rhGH in children with GHD. Key outcomes, including GH and IGF1 levels, growth, AEs, and immunogenicity were assessed. The results showed that lonapegsomatropin was as effective as daily rhGH (Genotropin^®^, Pfizer, New York, NY, USA) at the dose of 0.03 mg/kg/day. The annualized mean HV for the three lonapegsomatropin doses showed no statistically significant differences compared to Genotropin^®^ (ranging from 11.9 cm to 13.9 cm for lonapegsomatropin vs. 11.6 cm for Genotropin^®^), with GH levels within the physiological range and IGF1SD score normalized. Regarding safety, tolerability, and immunogenicity, AEs reported in the lonapegsomatropin cohorts were generally mild to moderate, and most were not considered related to the study drug. Injection site reactions were similar to those observed with daily rhGH, with no cases of lipoatrophy or nodule formation. No neutralizing anti-GH binding antibodies were detected in the lonapegsomatropin groups, indicating a low immunogenic potential and immunogenicity frequency comparable to daily rhGH. Due to the lipolytic effect of GH, the mean body mass index (BMI) SD score did not change significantly during lonapegsomatropin administration, whereas a slight decrease was observed in the Genotropin^®^ group. This modest decrease in patients in the Genotropin^®^ group was measured from slightly higher mean BMI values at baseline.

The heiGHt trial, a Phase 3 study, assessed the safety, tolerability, and efficacy of lonapegsomatropin administered once-weekly in children with GHD, compared to daily rhGH (NCT02781727) (33). Similar to endogenous somatropin, GH released from lonapegsomatropin is likely to be distributed into peripheral tissues and interact with GH receptors. In this study, patients treated with either lonapegsomatropin or daily rhGH showed comparable IGF1/IGFBP-3 ratios, maintaining the relationship between IGF1 and its binding protein at physiological levels. In this study, patients who were randomly assigned to receive lonapegsomatropin 0.24 mg/kg/week for 52 weeks showed superiority over daily rhGH (administered at an equivalent weekly dose) in terms of annualized HV and a statistically greater change in height SD score from baseline. However, both treatments showed similar bone age/chronological age ratios, as well as safety, tolerability, and immunogenicity (33). In the fall of 2021, lonapegsomatropin became the first once-weekly treatment approved by the FDA since somatropin (Nutropin Depot^®^). It was approved for once-weekly treatment in children ≥1 year who weigh ≥11.5 kg and have inadequate secretion of endogenous GH.

##### 3.1.5.2 PEGylated somatropin

Unlike lonapegsomatropin, which is transitively linked to PEG, PEGylated somatropin is an irreversibly PEGylated long-acting GH (Jintrolong^®^, GeneScience Pharmaceuticals, a subsidiary of Hualan Biological Engineering Inc., Shijiazhuang, Hebei Province, China) developed as an alternative to daily rhGH for weekly injection. The safety, tolerability, and pharmacokinetic of PEGylated somatropin have been demonstrated through comparative analyses in both healthy adult subjects (73) and children with GHD (74). In PEGylated somatropin structure, the amino group of rhGH is conjugated with a branched PEG molecule (40 kDa). This conjugation enhances protein stability, reduces antigenicity and non-specific absorption, decreases renal clearance, and prolongs the elimination half-life of rhGH (35). PEGylated somatropin is marketed in China for the treatment of children with GHD.

In Phase 2 and Phase 3 multicenter randomized trials treatment with PEGylated somatropin at a dose of 0.2 mg/kg/week for 25 weeks was shown to be non-inferior in terms of efficacy and safety when compared to daily rhGH (0.25 mg/kg/week) for the treatment of children with GHD (35). The growth-promoting effects of PEGylated somatropin were consistent with those observed for other long-acting GH formulation. Notably, PEGylated somatropin demonstrated greater efficacy in increasing HV, height SD score, and IGF1 SD score at a lower weekly dosage compared to daily rhGH. Most patients treated with PEGylated somatropin in this study had serum IGF1 levels within the normal range. Only a small percentage of patients showed elevated IGF1 levels, which would typically necessitate a dose reduction in clinical practice. In a 24-month follow-up study enrolling Chinese children with GHD demonstrated comparable efficacy and safety than daily rhGH (36).

In terms of safety, PEGylated somatropin was well tolerated, with an AE profile consistent with other long-acting GH formulations (75). However, a higher prevalence of peripheral edema was observed, which was not reported in studies involving LB03002 or somatropin in pediatric patients with GHD. This peripheral edema was generally mild and did not lead to treatment discontinuation. During the study, only four patients (three receiving PEGylated somatropin and one receiving daily rhGH) reported hypothyroidism. No cases of injection-site lipoatrophy or development of anti-GH antibodies were observed in any of the treated patients. The absence of lipoatrophy in patients receiving PEGylated somatropin or daily rhGH may be attributed to the reduced number of injections at the same site and rapid s.c. absorption of PEGylated somatropin. The lack of anti-GH antibodies in these groups may be due to the short duration of the trial (31) or to the low immunogenicity and antigenicity resulting from the PEGylation process.

In China, PEGylated somatropin is in the market for GHD children. However, all long-acting PEGylated GH formulations were withdrawn in Europe and the US. In response to this, a report by the European Medicines Agency (EMA) addressed potential safety concerns related to the use of PEGylated drug products in pediatric patients (75).

##### 3.1.5.3 Somavaratan

Somavaratan (VRS-317) (Eagle, Versartis, Inc., Redwood City, CA, USA) is a fusion protein produced in *Escherichia coli*, designed to reduce the clearance of rhGH from the body (76). In somavaratan, rhGH is bound to long chains of natural hydrophilic amino acids (XTEN), which enhance the drug’s potency by prolonging its exposure at the target site. The binding with the N-terminal XTEN sequence, increases the hydrodynamic size of the rhGH, reducing glomerular filtration, while the addition of a C-terminal XTEN sequence decreases receptor-binding, thus reducing receptor-mediated clearance.

In adults with GHD, somavaratan exhibited an elimination half-life that was 30–60 times longer than that of rhGH, allowing for injections at intervals as long as monthly. This extended half-life resulted in sustained IGF1 responses that outlasted those seen with daily rhGH administration (77). In prepubertal children with GHD, somavaratan significantly impacted serum IGF1 levels and HV, with no significant differences observed between monthly, twice-monthly, or weekly dosing regimens (47).

During the 30-day dose-finding phase of the study, patients received single ascending doses of somavaratan (0.8, 1.2, 1.8, 2.7, 4.0, or 6.0 mg/kg) (47). Following this, the drug was randomly administered on a weekly (1.15 mg/kg), twice monthly (2.5 mg/kg), or monthly (5.0 mg/kg) basis for a period of 6 months (47). The pharmacokinetic of somavaratan was dose-dependent, and serum IGF1 levels increased in a dose-dependent manner, with sustained responses observed across all dosing intervals. A single dose of somavaratan resulted in IGF1 responses lasting up to one month, with no accumulation of the drug or IGF1 observed following repeated dosing.

Annualized mean HV was similar across the different dosing schedules, with values of 7.86 ± 2.5 cm/year for monthly dosing, 8.61 ± 2.7 cm/year for twice-monthly dosing, and 7.58 ± 2.5 cm/year for weekly dosing. Overall, all tested doses of somavaratan were well tolerated, with AEs being mild and transient. No cases of injection site lipoatrophy or nodule formation were reported (47).

However, in a subsequent phase 3 clinical trial, somavaratan failed to demonstrate non-inferiority to daily recombinant human GH in terms of annualized height velocity. Consequently, its clinical development was discontinued, and the drug has not received regulatory approval.

## 4 Concluding remarks

Long-acting GH formulations represent a significant advancement in the management of pediatric growth hormone deficiency, primarily by reducing injection frequency and potentially improving treatment adherence. Current evidence indicates that newer long-acting agents, including Somapacitan, Somatrogon, and Lonapegsomatropin, demonstrate efficacy and safety profiles comparable to daily recombinant human GH, without the emergence of unexpected adverse events.

However, important uncertainties remain. These include the long-term safety of sustained GH and IGF1 exposure, potential metabolic effects, and the impact of prolonged pharmacokinetic profiles on physiological hormone dynamics. In addition, issues related to cost-effectiveness, patient adherence in real-world settings, and long-term outcomes require further investigation.

Overall, while long-acting GH formulations offer a promising alternative to daily therapy, their use may be particularly advantageous in patients with poor adherence to daily injections or those experiencing significant treatment burden. Conversely, caution may be warranted in specific populations, including very young children, patients requiring frequent dose adjustments, and those in whom close monitoring of IGF-1 levels is critical. In addition, the use of long-acting GH in non-GHD conditions or in patients with complex comorbidities requires further evidence. Continued long-term and real-world studies are essential to better define the optimal patient selection and to fully establish the clinical role and safety profile of these therapies.
